# Tensile properties of millimeter-long multi-walled carbon nanotubes

**DOI:** 10.1038/s41598-017-10279-0

**Published:** 2017-08-25

**Authors:** Hyung-ick Kim, Mei Wang, Stephanie K. Lee, Junmo Kang, Jae-Do Nam, Lijie Ci, Jonghwan Suhr

**Affiliations:** 1 Korea Institute of Industrial Technology, 25 Yeonkkot-ro, 165 beon-gil, Jeongchon-myeon, Jinju-si, Gyeongsangnam-do, 52845 Republic of Korea; 2 Department of Energy Science, Sungkyunkwan University, 2066, Seobu-ro, Jangan-gu, Suwon-si, 16419 Republic of Korea; 3 Department of Materials Science and Engineering, Northwestern University, Evanston, Illinois 60208 United States; 4 Department of Polymer Science and Engineering, Sungkyunkwan University, 2066, Seobu-ro, Jangan-gu, Suwon-si, 16419 Republic of Korea; 5 SDU & Rice Joint Lab for Carbon Nanomaterials, Key Laboratory for Liquid-Solid Structural Evolution & Processing of Materials (Ministry of Education), School of Materials Science and Engineering, Shandong University, Jinan, 250061 China; 6 Department of Mechanical Engineering, Sungkyunkwan University, 2066, Seobu-ro, Jangan-gu, Suwon-si, 16419 Republic of Korea

## Abstract

There have been a number of theoretical and experimental studies on tensile properties of carbon nanotubes (CNT), reporting the Young’s modulus of the individual CNT up to 1 TPa. Although CNT shows the promise to be used as reinforcement in a high modulus/strength composite material, it exhibits quite disappointing in terms of modulus or strength. Along with recent advance in CNT growth technique, we will be able to directly measure tensile properties of millimeter-long MWCNTs. This study firstly tackles the direct measurement of the tensile properties of millimeter-long MWCNTs that can be used as reinforcement in a composite system. A carefully designed tensile testing technique for the MWCNTs is developed, which allows us to obtain more accurate and reliable measured values. The average tensile strength and Young’s modulus of the CNTs investigated in this study are measured to be 0.85 GPa and 34.65 GPa, respectively. Also, this work statistically investigates the effect of the CNT dimensions including length, diameter and volume on the tensile properties. To the best of our knowledge, this is the very first report on the tensile properties of macroscopically long and continuous CNTs.

## Introduction

Since its discovery, carbon nanotube (CNT) is believed to be the strongest and stiffest materials because it may be thought as a rolled-up sheet of graphite. Theoretical predictions and experimental observations have been carried out on the tensile properties of CNTs^1–8^. For instance, J. R. Xiao *et al*. predicted that the tensile strength of single-walled CNTs (SWCNTs) is 94–126 GPa by an analytical molecular structural mechanics model and M. Rossi, *et al*. reported that the Young’s modulus of SWCNTs is around 0.915 TPa^9, 10^. As an evidence, R. S. Ruoff analyzed a single SWCNT using a tensile loaded rope between an atomic force microscope (AFM) tip and a SWCNT “paper”, showing that the Young’s modulus of SWCNT is around 1 TPa^11^. By the same method, Ruoff also investigated the tensile property of multi-walled CNTs (MWCNTs) which are attached between two AFM tips. It was observed that the tensile strength of an individual MWCNT is 63 GPa and its Young’s modulus is 270–950 GPa^12^. It should be noted that the length of CNTs investigated in the aforementioned studies are found to be only at most several micron.

Undesirably, for the CNT reinforced composites, the outstanding mechanical properties of individual CNTs have not been translated into the nanocomposites^13^. Certainly it attributes the dispersion and waviness of the CNTs in a matrix to the poor modulus and strength of composites. Although the contributions might be significant, this could be also an indication of the poor mechanical properties of the CNT as reinforcement in a composite system. In fact, due to the nanoscale dimensions and the corresponding unavoidable assumptions in measurement, it always presents significant challenges to accurately measure and determine the mechanical properties. Often, the theoretical values of the Young’s modulus and tensile strength don’t take into account the defects in CNT, which are generated during the synthesis. The defect properties of CNT will play a significant role in determining their mechanical properties^14^, as well as other material properties including electrical and thermal transport; if the CNT can be synthesized with defect-free, the tensile strength and modulus of CNT would reach the theoretically obtained values. The amount and properties of defects in CNT are crucially related to the synthesis process. Although the presence of CNT defects will dominate the mechanical properties, a little attention seems to be made to consider the effect on the properties particularly when the CNTs are utilized as reinforcement in a composite. Apparently, no reports are available on the direct measurement of the intrinsic tensile properties of the CNT as a constituent in a composite system. Here, it is critically important to measure the tensile properties of the CNT in order to properly design the composite materials.

The tensile properties of the spun CNT fibers have also been investigated^15^. In general, the CNT fibers are spun by typically three processes, which is, spun from CNT solutions (wet-spun)^3, 16^, CNT aerogels (aerogel-spun)^17, 18^, and CNT arrays (forest-spun)^19–21^. However, the CNT fibers are bonded at van der Waals force in length direction, which will result in slippage between nanotubes while experiencing loadings. Our literature survey indicates that so far, the tensile properties of macroscopically long and continuous CNT have not been investigated. With recent advance in chemical vapor deposition (CVD) method for vertically aligned MWCNT (VAMWCNT) forests in our earlier work, up to several centimeter-long MWCNTs can be synthesized^22, 23^.

Here we study an experimental investigation on the tensile properties of millimeter-long VAMWCNTs which are synthesized through the aforementioned CVD technique. The tensile strength and Young’s modulus are characterized with respect to CNT lengths, diameters, and volumes. The structure-properties relationship are also investigated via statistical analysis. To the best of our knowledge, this is the very first report on the direct measurement of the mechanical properties of the macroscopically long and continuous CNT.

## Results and Discussion

**Figure 1:**
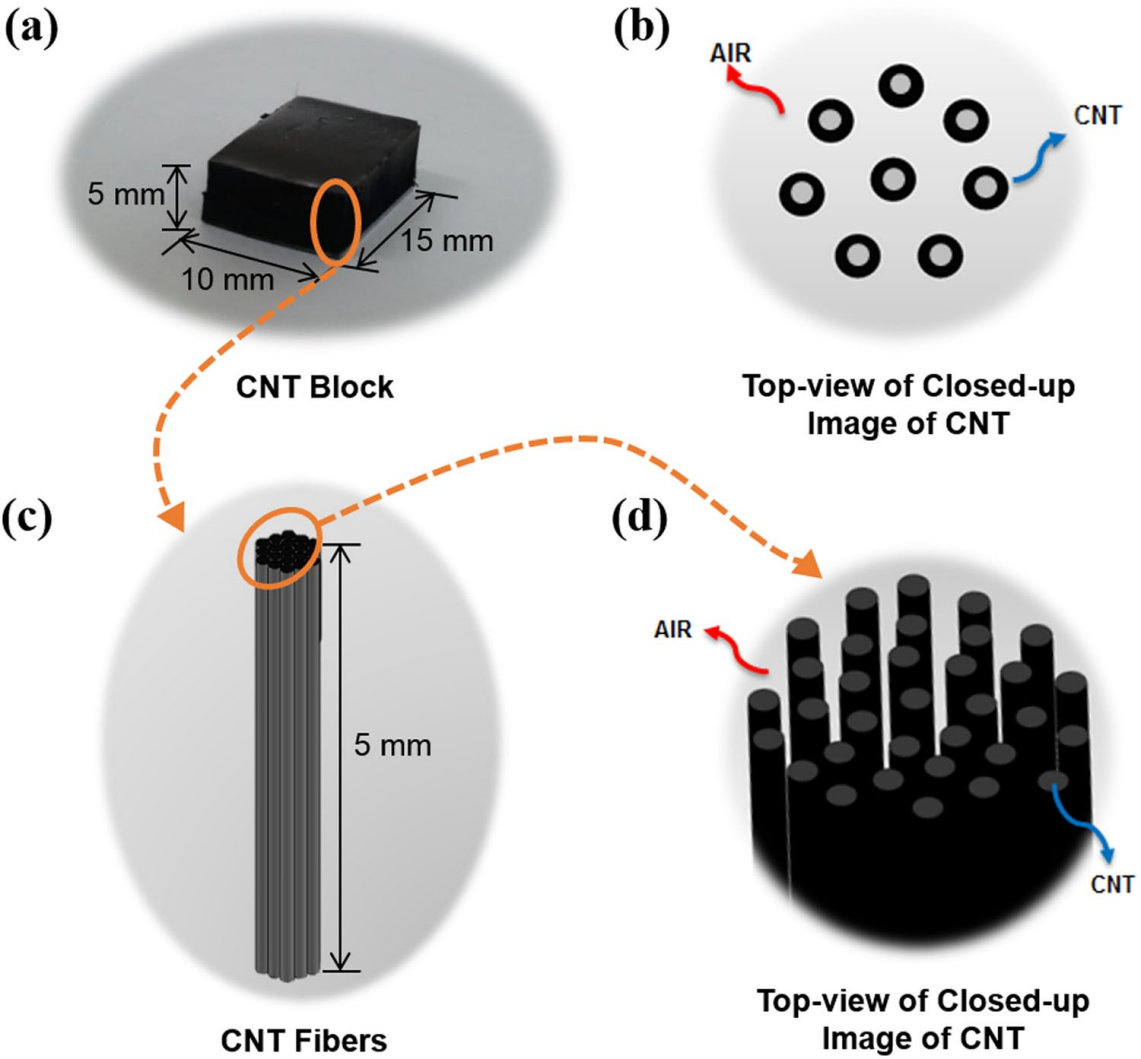
A schematic representation of millimeter-long vertically-aligned MWCNT.

The MWCNTs used in this study are synthesized by the CVD method reported previously^22^. A developed Fe (1 nm)/Al_2_O_3_ (30 nm)/SiO_2_ (300 nm)/Si (500 μm) substrate with the size of 10 × 15 mm^2^ is used as the catalyst/substrate. In order to achieve several millimeter-long VAMWCNTs, we design the CNT growth by controlling the catalyst thickness with a 1 nm Fe catalyst layer adopted in the CNT growth process. With this CNT growth technique, up to a few centimeter-long vertically aligned MWCNTs can be fabricated which allows the measurement of intrinsic CNT tensile properties. Figure 1 illustrates how the millimeter-long CNT samples can be obtained from the CVD grown VAMWCNT forest for the tensile testing and Supplementary Figure S1 shows the top-view of closed-up SEM image of CVD grown CNT bundles. The CNTs specimens in this study are directly drawn out from the cuboid CNT forest by carefully manipulating and stripping with tweezers. These CNTs have a high aspect ratio and large surface areas, therefore, they intend to get together and be assembled into bundles due to the van der Waals interaction between tubes^24^. And this axially compressed MWCNTs are considering as a cylindrical shell continuum, which has a circular cross-sectional area^25^. The CNTs would have wavy structure in their length direction, and correspondingly the air space inside the CNT would be fluctuated. However, we assumed that the CNTs are straight in their length since such effect is negligible. Moreover, the CNT bundles consist of double-walled and 3.8 nm of the average inner diameter with 0.34 nm of the gap between neighboring CNT walls^22^.

**Figure 2:**
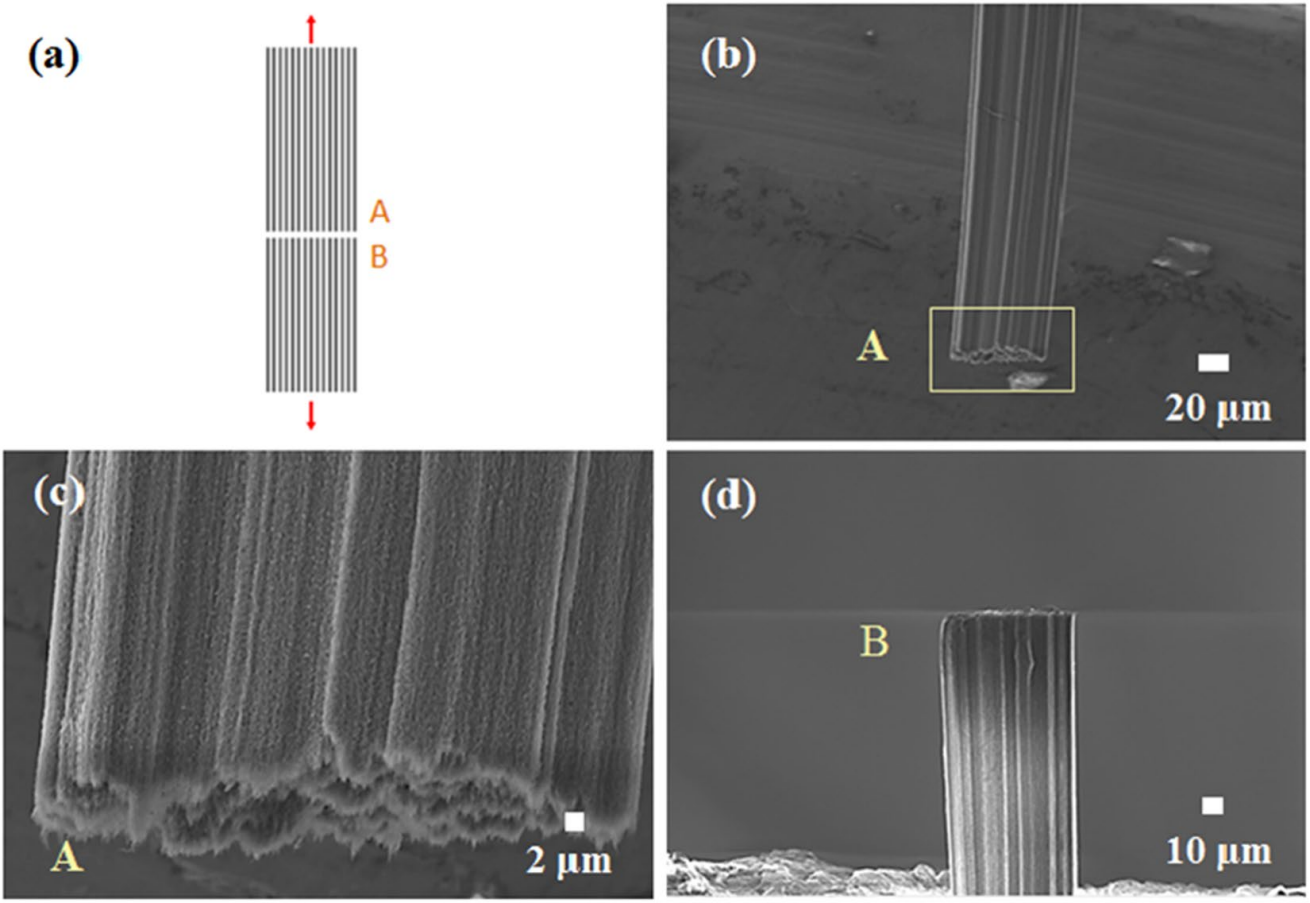
A schematic and SEM images of MWCNTs. (**a**) A schematic illustrating the tensile loading and nanotube specimen. (**b**) SEM image of the fracture morphology of the CNT specimen (Part A) after tensile testing. (**c**) SEM image showing the fracture surface morphology (Part A). (**d**) SEM image of the fracture morphology of the CNT specimen (Part B) after tensile testing.

After tensile testing as illustrated in Fig. 2a, the fracture morphology of the MWCNTs samples are examined by using scanning electron microscope (SEM). Figure 2b,c and d show the SEM images of the fracture morphology of the MWCNTs specimen tested in the study. Based on our morphology examination, the CNT bundles were found to have a round shaped cross-section with a diameter of approximately 60 μm. Virtually all the nanotubes are still aligned in their length direction after the testing.

It is quite interesting to see that the tensile fracture behavior of the millimeter-long CNT samples appears to be very similar to the failure in single crystal-like materials. It is observed that the fracture surfaces seem to be more or less in a particular plane and also perpendicular to the nanotube length direction. The fracture morphology characterization indicates that the failure initiation site on the fracture surfaces is not clearly identified and located in this study. However, obviously, the initial crack would begin where a great deal of defects exist along the nanotube length direction. The maximum stress will be then developed at the initial failure site, and the resultant crack will continue to propagate by cutting through the nanotubes in the transverse direction. In this way, the fracture cross sections, as seen in Fig. 2, are formed in a perpendicular to the nanotube length direction. As a consequence, this can clearly indicate that the defects of the CNTs are mainly responsible for the observed tensile failure behavior. Also note that the transverse shrinking phenomenon previously observed in spun CNT fibers^15^ is not seen in the millimeter-long continuous MWCNT investigated in this study.

**Figure 3:**
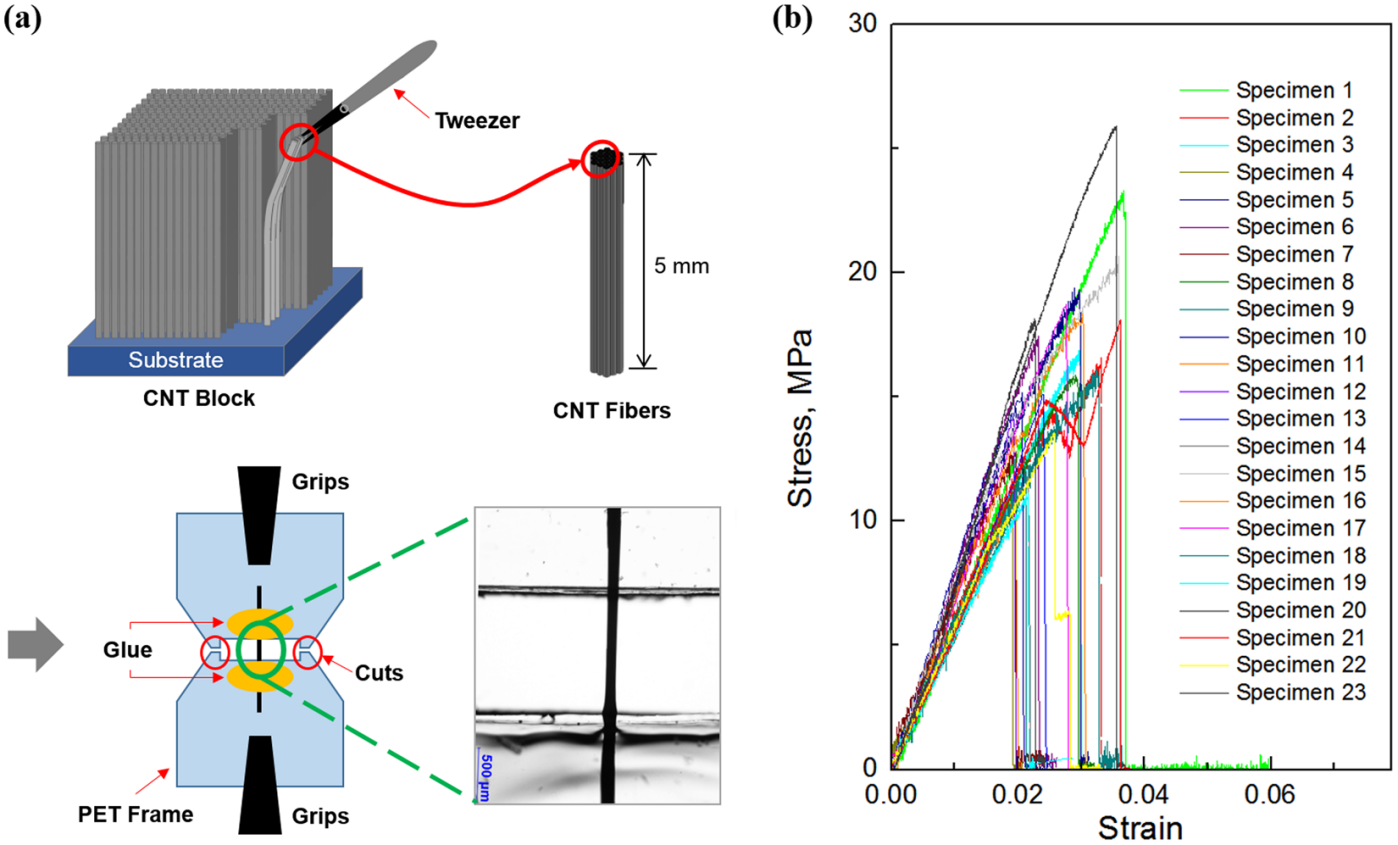
(**a**) A schematic of nanotube tensile testing set-up and an optical microscopic image of the MWCNT mounted on the frame (1 mm in length and 98 μm in diameter). (**b**) The stress-strain curves of the millimeter-long vertical aligned MWCNTs. (The length and diameter of specimens are in ranges from 0.75 to 2.15 mm, and 25 to 107 μm, respectively.)

Due to nanoscale dimensions and high specific surface energy, it is very difficult to mount the CNTs onto a specimen frame and fixture for tensile testing. Following the typical standard test methods for the single fiber testing doesn’t ensure consistency and repeatability for each testing. Here, we carefully designed a test frame by using a polyethylene terephthalate (PET) with an appropriate dimension. The millimeter-long MWCNTs samples were directly pulled out from CNT block using tweezers, then the samples with the shaped of nanotube bundle were laid across the pre-designed frame and each end of the samples was attached using an adhesive. Prior to the mounting the specimen for the tensile testing, the length and diameter of each specimen were measured and characterized using an optical microscope (Fig. 3a). Note that the length (L) and diameter (d) of the specimens investigated in this study are in ranges from 0.75 to 2.15 mm, and 25 to 107 μm, respectively. The frame was properly mounted onto micro-pneumatic grips, and then each side of the frame was carefully cut before applying tensile load.

Figure 3b shows the typical stress-strain behaviors of millimeter-long MWCNT samples. It exhibits typical behaviors of brittle materials, showing a quite clear linear elastic region and ranging from 0.2% to 0.4% in elongation at break. Noticeably, the macroscopically measured data of tensile strength and Young’s modulus of the MWCNT samples are an order of magnitude less than those of individual single-walled or multi-walled carbon nanotubes with microscale length^9–12^. The tensile strength is found to be up to 23.32  MPa, while the greatest Young’s modulus is determined to be only around 830  MPa. Such lower modulus and strength should be largely attributed to the free space between nanotubes and along the length. As illustrated in Fig. 1b, the cross-section of the MWCNTs sample is mainly occupied by air space between neighboring nanotubes. In addition, F. Hill, *et al*. reported that tensile properties of continuous millimeter-scale carbon nanotube fibers are also determined by densification of CNT fibers^26^. In calculation of the stress presented in Fig. 3b, we assume the MWCNT sample has a solid and uniform cross-section with a constant diameter (d) along the length.

Since our interest focuses on the measured intrinsic tensile properties of the millimeter-long MWCNT, it should be important to define the net cross-sectional area of MWCNTs samples. For our convenience, the density of CNT is considered to be the same as the one of graphite which is 2.25 g/cm^3^. The density of air, ρ_air_ is 1.29 × 10^−3^ g/cm^3^. The total density of both pure CNT and air in the CNTs, ρ_all_ can be measured and also presented in the Eqn. 1 as follows:

1 $${\rho }_{all}=\frac{{\rho }_{CNT}{V}_{CNT}+{\rho }_{air}{V}_{air}}{{V}_{all}}=\frac{{\rho }_{graphite}AL+{\rho }_{air}\frac{1}{4}\pi {d}^{2}L-AL}{\frac{1}{4}\pi {d}^{2}L}$$

where the ρ_all_ is determined to be 0.045 g/cm^[3 27^. Here, A indicates the net area of CNTs in the cross-section of the MWCNT bundle and L denotes the length of CNTs. V_CNT_ equals to A × L. The value of A can now be back-calculated from Eqn. 1. Thus the air fraction of CNT (η) can be calculated as follows:

2 $${\rm{\eta }}=1-\frac{A}{\frac{1}{4}\pi {d}^{2}}=\,\frac{{\rho }_{graphite}-\,{\rho }_{all}}{{\rho }_{graphite}-\,{\rho }_{air}}$$

where the η is calculated to be 98.1%.

**Figure 4:**
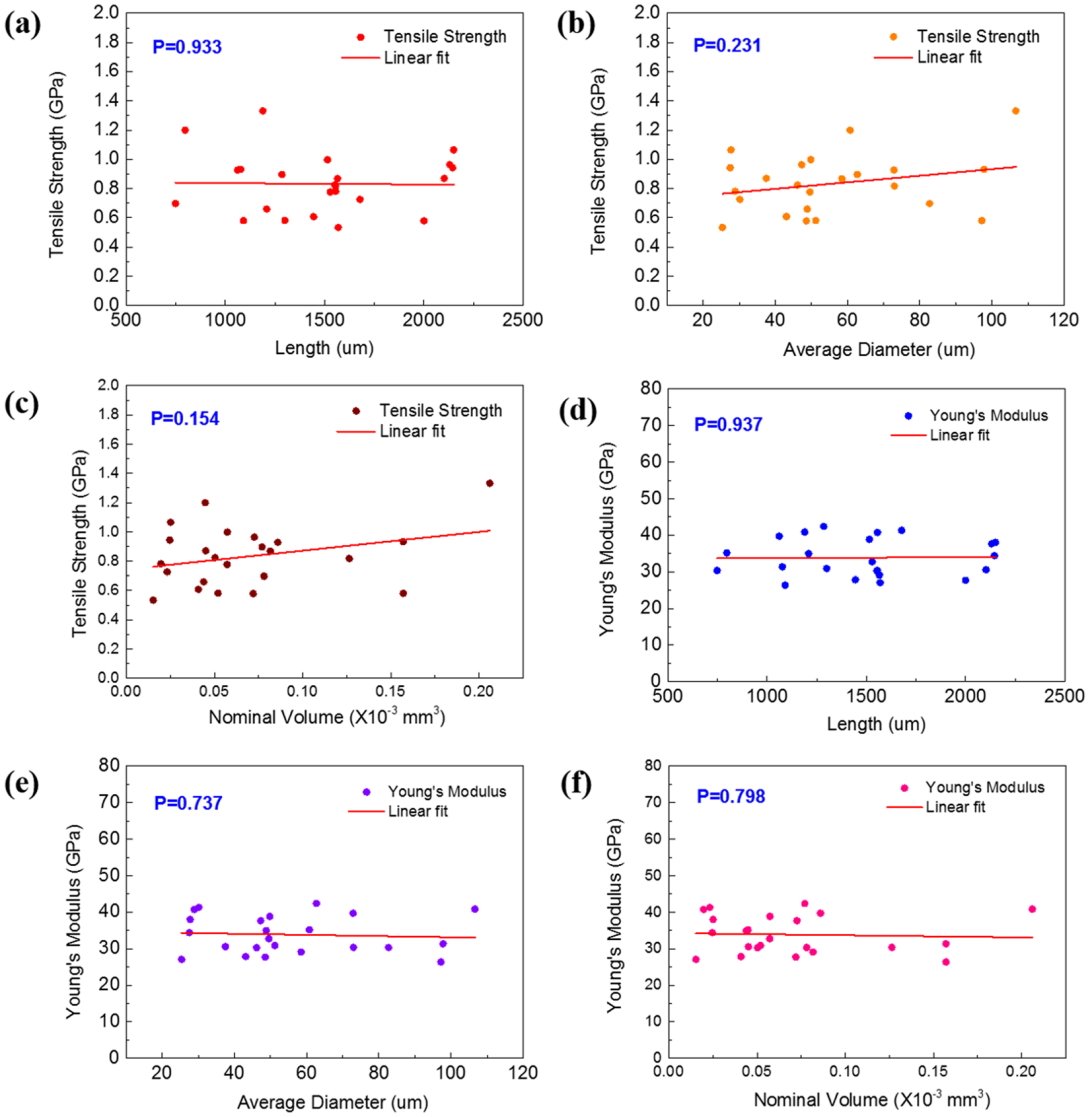
The tensile strength values with different length (**a**), diameter (**b**), and volume (**c**) of MWCNTs, and Young’s modulus values with different length (**d**), diameter (**e**), and volume (**f**). Significance level is defined as P_cr_ = 0.05.

In order to evaluate the intrinsic tensile properties of the continuous nanotubes, the stress needs to be re-calculated with the aforementioned nominal cross-sectional area for the same data of the measured loads and displacements^15^. Taking into account the free space occupied by air in the MWCNT bundles, Fig. 4 compares their intrinsic tensile strength and Young’s modulus with respect to CNT microstructure (diameter, length, and volume). A noticeable feature of the results is that the intrinsic strength and modulus are more than an order of magnitude greater than those seen in Fig. 3b. The intrinsic tensile strength and Young’s modulus of the continuous CNTs are 0.85 GPa and 34.65 GPa in average, respectively. Notably, the tensile properties of the CNTs are a factor of dozens of times less than the Young’s modulus and tensile strength of the individual SWCNT or MWCNT reported earlier^28–30^.

It is believed that the tensile strength would be mainly determined by the existence of intrinsic defects in the CNTs^1^. Given that the number of the defects could be proportional to the greater dimensions including the length and diameter of the CNT bundles, it might be important to compare the tensile strength and Young’s modulus with respect to length, diameter and volume of CNTs specimens (Fig. 4). Regarding to it, linear regression analysis is carried out and the p-value (P) is calculated as shown in Fig. 4 to study the relationship between tensile strength/Young’s modulus and CNT dimensions (length, diameter and volume). A low P which is lower than critical p-value (P_cr_ = 0.05) indicates that the data align linearly. Interestingly, linear regression analysis indicates no clear linear relationship between tensile strength with CNT length (P = 0.933), diameter (P = 0.231), or volume (P = 0.154). Likewise, no linear relationship is observed for the intrinsic Young’s modulus with respect to the CNT length (P = 0.937), diameter (P = 0.737), or volume (P = 0.798). These results confirm that both tensile strength and Young’s modulus are independent of the CNT length, diameter and volume. In fact, according to the definitions of stress and strain, the tensile properties of any materials should be independent of the geometry and dimensions of test specimens. As a consequence, it can be concluded that the existence of the intrinsic defects in CNTs mainly affects their intrinsic tensile strength and Young’s modulus, not the number of the defects. The Raman spectra shows an obvious D peak at 1350 cm^−1^ and the *I*_*D*_/*I*_*G*_ of the MWCNTs were determined to be up to 0.5. The defect density in the MWCNTs investigated in this study is found to be much higher than the hexagonal crystal structure such as graphite, according to the high intensity of D peak of CNT^31^. More details on defects of the MWCNTs, which were synthesized by the same growth technique with this present study can be found in our earlier work^22^. Since the intrinsic defects generated from the CNT synthesis, which is particularly true for the CNT mass productions such as the chemical vapor deposition method, seems to be inevitable, it is necessary to carefully consider and accurately obtain the tensile properties of the CNTs when to use as reinforcement in a composite system.

## Conclusions

In this study, a carefully designed tensile testing technique for macroscopically long MWCNTs was developed and the intrinsic tensile properties of the CNTs were directly measured and accurately determined. The tensile strength and Young’s modulus of the continuous MWCNT bundles are found to be dozens of magnitude lower than those of the individual MWCNT which were determined by taking into accounts the abundant air space between nanotubes along the length.

However, the intrinsic tensile strength and Young’s modulus appear to be far less than those obtained from the numerical predictions or theoretical models. Such poor properties can result from the existence of intrinsic defects in the CNTs. Our statistical analysis results also confirmed that the intrinsic tensile strength and Young’s modulus have no linear-relationship with the CNT dimensions including length, diameter and volume. Consequently, this could conclude that the existence of the defects mainly affects their tensile properties, probably not the number of the defects in the CNTs.

To the best of our knowledge, this study firstly reports the direct measurement of the intrinsic tensile properties of millimeter-long MWCNTs. It will be critically important to understand and accurately obtain the mechanical properties of the nanoscale fillers such as CNT or graphene for better design and optimization of the nanoscale reinforcement composites.

## Methods

### Materials preparation

The millimeter-long VAMWCNTs were synthesized by a CVD process on the substrate of Fe/Al_2_O_3_/SiO_2_/Si. The Fe catalyst was deposited on the Al_2_O_3_/SiO_2_/Si substrate by the e-beam evaporation technique with the thickness of 1 nm. After the deposition, the Fe/Al_2_O_3_/SiO_2_/Si substrate was cut into pieces (10 × 15 mm^2^) with the dicing saw (DASCO:DAC552). The He (180 sccm), H_2_ (55 sccm) and C_2_H_4_ (25 sccm) gases were used as the carrier gas, and carbon source, respectively. The growth process was conducted on 760 °C for 8 hours.

### Characterization

A developed fabrication method was used to prepare specimens to characterize the tensile properties of carbon nanotubes^15^. To achieve sufficient statistical power, 23 MWCNT specimens with different diameter, length and volume were tested. Each testing frame was made from the PVC with a uniformly sized rectangular hole (7.5 mm × 2.5 mm) in the center (20 mm × 30 mm). Silver paste was place on both top and bottom edges of the center hole and it cured at a room temperature for a day. Then, the continuous carbon nanotubes were drawn from the carbon nanotubes bundles and placed on top of the rectangular hole, where the silver paste was placed. Only one end of the carbon nanotubes was glued to the top edge while clamping to the tensile machine. Prior to testing, a guide was used to align the specimen and cut the frame along the sides to insulate the MWCNTs from the testing frame. The displacement-controlled load frame (Instron 5848 Micro Tester), which was connected with a micropneumatic grip (Instron 54851B), was used to test the mechanical characterizations of the MWCNTs. The 5 N load cell and a 20 μm/min tensile testing speed were applied to gain accurate measurements. The diameter and length of the CNT specimens were measured with the optical microscope and SEM^32–36^.

## Electronic supplementary material

Supplementary Information

## References

[1] Ruoff RS, Qian D, Liu WK (2003). Mechanical properties of carbon nanotubes: theoretical predictions and experimental measurements. C. R. Physique..

[2] Koziol K (2007). High-performance carbon nanotube fiber. Science..

[3] Dalton AB (2003). Super-tough carbon-nanotube fibres. Nature..

[4] Miaudet P (2005). Hot-drawing of single and multiwall carbon nanotube fibers for high toughness and alignment. Nano Lett..

[5] Huang L, Cao D (2012). Mechanical properties of polygonal carbon nanotubes. Nanoscale..

[6] Roberts GS, Singjai P (2011). Joining carbon nanotubes. Nanoscale..

[7] Shen C, Brozena AH, Wang Y (2011). Double-walled carbon nanotubes: challenges and opportunities. Nanoscale..

[8] Nessim GD (2010). Properties, synthesis, and growth mechanisms of carbon nanotubes with special focus on thermal chemical vapor deposition. Nanoscale..

[9] Xiao JR, Gama BA, Gillespie JW (2005). An analytical molecular structural mechanics model for the mechanical properties of carbon nanotubes. Int. J. Solids Struct..

[10] Meo M, Rossi M (2006). Prediction of Young’s modulus of single wall carbon nanotubes by molecular-mechanics based finite element modelling. Compos. Sci. Technol..

[11] Yu MF, Files BS, Arepalli S, Ruoff RS (2000). Tensile loading of ropes of single wall carbon nanotubes and their mechanical properties. Phys. Rev. Lett..

[12] Yu MF (2000). Strength and breaking mechanism of multiwalled carbon nanotubes under tensile load. Science..

[13] Domun N (2015). Improving the fracture toughness and the strength of epoxy using nanomaterials–a review of the current status. Nanoscale..

[14] Schulz M (2012). Speeding up artificial muscles. Science..

[15] Wu AS, Chou TW, Gillespie JW, Lashmore D, Rioux J (2012). Electromechanical response and failure behaviour of aerogel-spun carbon nanotube fibres under tensile loading. J. Mater. Chem..

[16] Vigolo B (2000). Macroscopic fibers and ribbons of oriented carbon nanotubes. Science..

[17] Li YL, Kinloch IA, Windle AH (2004). Direct spinning of carbon nanotube fibers from chemical vapor deposition synthesis. Science..

[18] Zhong XH (2010). Continuous multilayered carbon nanotube yarns. Adv. Mater..

[19] Jia J (2011). A comparison of the mechanical properties of fibers spun from different carbon nanotubes. Carbon..

[20] Zu M (2012). Characterization of carbon nanotube fiber compressive properties using tensile recoil measurement. ACS Nano..

[21] Zhang M, Atkinson KR, Baughman RH (2004). Multifunctional carbon nanotube yarns by downsizing an ancient technology. Science..

[22] Kim H (2011). Synthesis of ultra-long super-aligned double-walled carbon nanotube forests. J. Nanosci Nanotechnol..

[23] Kim H (2011). Flow-dependent directional growth of carbon nanotube forests by chemical vapor deposition. Nanotechnology..

[24] Kanoun O (2014). Flexible Carbon Nanotube Films for High Performance Strain Sensors. Sensors..

[25] He XQ (2005). Modeling of van der Waals force for infinitesimal deformation of multi-walled carbon nanotubes treated as cylindrical shells. International Journal of Solids and Structures..

[26] Hill FA (2013). Enhancing the Tensile Properties of Continuous Millimeter-Scale Carbon Nanotube Fibers by Densification. ACS Appl. Mater. Interfaces..

[27] Li Y, Kang J, Choi JB, Nam JD, Suhr J (2015). Determination of material constants of vertically aligned carbon nanotube structures in compressions. Nanotechnology..

[28] Walters DA (1999). Elastic strain of freely suspended single-wall carbon nanotube ropes. Appl. Phys. Lett..

[29] Krishnan A, Dujardin E, Ebbesen TW, Yianilos PN, Treacy MMJ (1998). Young’s modulus of single-walled nanotubes. Phys. Rev. B..

[30] Demczyk BG (2002). Direct mechanical measurement of the tensile strength and elastic modulus of multiwalled carbon nanotubes. Mater. Sci. Eng., A..

[31] Collins PG (2010). Defects and disorder in carbon nanotubes. Oxford Handbook of Nanoscience and Technology: Materials: Structures, Properties and Characterization. Techniques.

[32] Zhu HW (2002). Direct synthesis of long single-walled carbon nanotube strands. Science..

[33] Sammalkorpi M (2004). Mechanical properties of carbon nanotubes with vacancies and related defects. Physical Review B.

[34] Mielke SL (2004). The role of vacancy defects and holes in the fracture of carbon nanotubes. Chemical Physics Letters.

[35] Hirsch A (2002). Functionalization of single-walled carbon nanotubes. Angew. Chem. Int. Ed..

[36] Eddesen TW, Takada T (1995). Topological and sp3 defect structures in nanotubes. Carbon.

