# Supplementary material for: Tensile properties of millimeter-long multi-walled carbon nanotubes
Source: Sci Rep. 2017 Aug 25;7:9512. doi: 10.1038/s41598-017-10279-0 (PMC5573399; doi:10.1038/s41598-017-10279-0)
Supplement: Supplementary file 1 — Supplementary Information [file 41598_2017_10279_MOESM1_ESM.pdf]

Tensile properties of millimeter-long multi-walled carbon nanotubes

Hyung-ick Kim[1], Mei Wang[2], Stephanie K. Lee[2], Junmo Kang[3], Jae-Do Nam[2,4], Lijie Ci[5] and Jonghwan Suhr[2,4,6,*]

[1]Korea Institute of Industrial Technology, 25 Yeonkkot-ro, 165 beon-gil, Jeongchon-myeon, Jinju-si, Gyeongsangnam-do, 52845, Republic of Korea
[2]Department of Energy Science, Sungkyunkwan University, 2066, Seobu-ro, Jangan-gu, Suwon-si, Republic of Korea
[3]Department of Materials Science and Engineering, Northwestern University, Evanston, Illinois, 60208, United States
[4]Department of Polymer Science and Engineering, Sungkyunkwan University, 2066, Seobu-ro, Jangan-gu, Suwon-si, Republic of Korea
[5]SDU & Rice Joint Lab for Carbon Nanomaterials, Key Laboratory for Liquid-Solid Structural Evolution & Processing of Materials (Ministry of Education), School of Materials Science and Engineering, Shandong University, Jinan, 250061, China
[6]Department of Mechanical Engineering, Sungkyunkwan University, 2066, Seobu-ro, Jangan-gu, Suwon-si, Republic of Korea

*Corresponding author: suhr@skku.edu

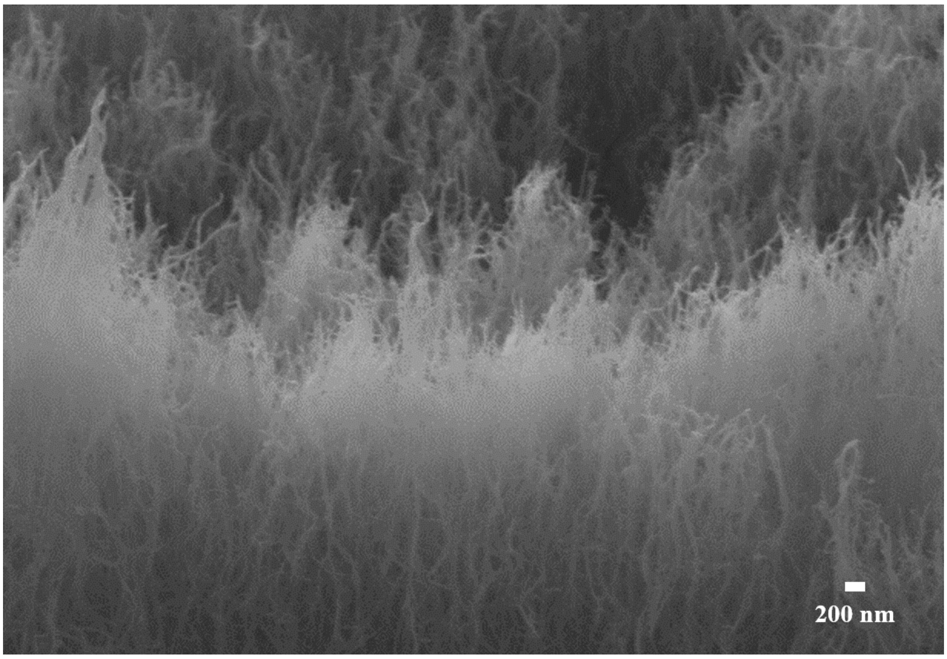

Supplementary Figure S1. The top-view of closed-up SEM image of CVD grown CNT bundles.
